# Central Administration of 5Z-7-Oxozeaenol Protects Experimental Autoimmune Encephalomyelitis Mice by Inhibiting Microglia Activation

**DOI:** 10.3389/fphar.2017.00789

**Published:** 2017-11-03

**Authors:** Lingli Lu, Xiuping Zhang, Huichun Tong, Wenlong Zhang, Pingyi Xu, Shaogang Qu

**Affiliations:** ^1^Department of Neurology, Shunde Hospital, Southern Medical University, Foshan, China; ^2^Department of Immunology, School of Basic Medical Sciences, Southern Medical University, Guangzhou, China; ^3^Department of Neurology, Zhujiang Hospital, Southern Medical University, Guangzhou, China; ^4^Teaching Center of Experimental Medicine, School of Basic Medical Sciences, Southern Medical University, Guangzhou, China; ^5^Department of Neurology, The First Affiliated Hospital of Guangzhou Medical University, Guangzhou, China

**Keywords:** 5Z-7-oxozeaenol, transforming growth factor β-activated kinase 1, experimental autoimmune encephalomyelitis, microglia, NF-κB, mitogen-activated protein kinase

## Abstract

Transforming growth factor β-activated kinase 1 (TAK1), a vital upstream integrator of multiple pro-inflammatory signaling pathways, mediates the production of pro-inflammatory cytokines, chemokines, and adhesion molecules. Investigations targeting TAK1 provide new therapeutic options for chronic inflammatory disorders, autoimmune diseases, and cancer. However, the role and mechanism of the TAK1 inhibitor 5Z-7-oxozeaenol in treating autoimmune demyelinating diseases remain unclear. This work aimed to identify whether 5Z-7-oxozeaenol exerts neuroprotective effects on experimental autoimmune encephalomyelitis (EAE) in mice. Here, we demonstrate that 5Z-7-oxozeaenol efficiently alleviates the symptoms of EAE by decreasing the levels of pro-inflammatory cytokines in splenocytes and central nervous system, diminishing the number of activated microglia and inhibiting the p38MAPK, JNK, and ERK signaling pathways. Furthermore, we demonstrate that administration during the symptomatic time window is required for 5Z-7-oxozeaenol efficacy. These results suggest that TAK1 inhibition may provide a potent approach toward treating autoimmune demyelinating diseases.

## Introduction

Multiple sclerosis (MS) is a chronic, inflammatory, autoimmune disease of the central nervous system (CNS) that is characterized by demyelination and axonal destruction. Although the etiopathogenesis of MS has not been well elucidated, a cascade of pathological events is known to be involved in its pathology, including the disruption of the blood–brain–barrier (BBB), the activation of microglia and the infiltration of lymphocytes into the CNS, eventually giving rise to demyelination and degeneration of axonal structures (Lassmann et al., 2012; Martín et al., 2012; Goldmann and Prinz, 2013; Ciccarelli et al., 2014).

The activation of microglia has been described in both acute and chronic stages of MS and in its mouse model, experimental autoimmune encephalomyelitis (EAE). In fact, activated microglia nodules are the hallmark of MS lesion formation (Singh et al., 2013). Microglia are innate immune cells in the CNS that belong to the mononuclear phagocytic family and serve to patrol and monitor the microenvironment of the parenchyma. The repertoire of functions of microglia in MS is nuanced, complex and not well understood, though it has been well established that microglia contribute significantly to the overall disease severity of MS. Overactive microglia are responsible for detrimental effects leading to profound neurologic impairments, accounted for, in part, by their roles in producing pro-inflammatory cytokines, nitrogen species, reactive oxygen, and proteolytic enzymes (Merson et al., 2010). They are the main sources of cytokines and chemokines during CNS autoimmune inflammation (Hanisch, 2002). They also upregulate major histocompatibility complex class II molecules and present self-antigens, which function to drive autoimmunity (Correale, 2014).

However, the response of microglia may vary widely depending on the pathologic environment. Microglia can exhibit M1 (a cytotoxic phenotype) or M2 (a neuroprotective phenotype) activated states. M1 microglia produce pro-inflammatory cytokines, such as IL-1β, TNF-α, IL-6, IL-12, and IL-23, as well as free radicals (Wang et al., 2015); M2 microglia mediate anti-inflammatory responses and promote tissue repair. The M1 phenotype of microglia can be activated by lipopolysaccharide (LPS), IFN-γ, or TNF-α (Schmitz and Chew, 2008). Prolonged activation triggers pro-inflammatory cascades via several signaling pathways, including the nuclear factor kappa B (NF-κB), p38 mitogen-activated protein kinase (p38MAPK), c-Jun N-terminal kinase (JNK), and extracellular signal-regulated kinase (ERK) pathways (Kaltschmidt et al., 1994; Shin et al., 2003; Waetzig et al., 2005; Merson et al., 2010). p38MAPK, JNK, and ERK are serine/threonine kinases of the mitogen-activated protein kinase (MAPK) family. Furthermore, sustained activation of the NF-κB signaling pathway in microglia/macrophages in LysMCreIκBαfl/fl mice aggravates the clinical signs of EAE and elevates the production of IL-1β, IL-6, and other pro-inflammatory cytokines (Ellrichmann et al., 2012). Pro-inflammatory cytokines, including TNF-α, IFN-γ, IL-6, and IL-1β, activate microglia and shift them toward a M1 cytotoxic phenotype, which potentiates the inflammatory response and contributes to tissue damage.

Transforming growth factor β-activated kinase 1 (TAK1), also known as the mitogen-activated kinase kinase kinase (MAP3K), is a common upstream integrator of NF-κB, p38MAPK, JNK, and ERK and can be activated by transforming growth factor-β, TNF-α, IL-1, LPS, and BCR/TCR (Ajibade et al., 2013). Activated TAK1 strongly elicits the expression of pro-inflammatory cytokines, chemokines, and adhesion molecules. Given the role of TAK1 as a vital regulator of inflammatory and immune signaling pathways, the suppression of TAK1 production has been explored as a therapeutic strategy for chronic inflammatory disorders, including autoimmune diseases and cancer (Sakurai, 2012). Selective TAK1 knock out in microglia has been reported to prevent the release of pro-inflammatory mediators and effector molecules in the EAE model, leading to significantly ameliorated CNS inflammation and reduced tissue damage (Goldmann et al., 2013). However, the use of a pharmacological TAK1 inhibitor in treating EAE has not been described.

5Z-7-oxozeaenol, a resorcylic acid lactone derived from fungus, is a powerful inhibitor of TAK1 (Ninomiya-Tsuji et al., 2003). Selective inhibition of TAK1 with 5Z-7-oxozeaenol is known to block pro-inflammatory signaling (Wu et al., 2013); however, the impact of 5Z-7-oxozeaenol on EAE has not been fully delineated. Herein, we assessed the effect and mechanism of 5Z-7-oxozeaenol on EAE mice. Our results demonstrate that 5Z-7-oxozeaenol can effectively alleviate the symptoms of EAE, reduce the inflammatory response and demyelination in the CNS, decrease the levels of IL-17A, IFN-γ, TNF-α, and IL-6, and reduce the number of activated microglia in the CNS by inhibiting the p38MAPK, JNK, and ERK signaling pathways. These results suggest that TAK1 inhibition may provide a potent approach toward treating autoimmune demyelinating diseases.

## Materials and Methods

### Animals

C57BL/6 female mice (19–22 g) were purchased from the Experimental Animal Center of Guangdong Province, China. All mice were fed in pathogen-free conditions with standard laboratory chow and water *ad libitum*. All experiments were approved and conducted in accordance with the guidelines of the Animal Ethics Committee of the Nanfang Hospital. Efforts were made to minimize the suffering of mice during experiments.

### Reagents

MOG_35-55_ (MEVGWYRSPFSRVVHLYRNGKCOOH; purity >98%) was purchased from Shanghai Science Peptide Biological Technology Co., ltd. Incomplete Freund’s adjuvant (F5506) and LPS (*Escherichia coli* O55:B5) were purchased from Sigma–Aldrich. *Mycobacterium tuberculosis* H37 Ra (231141) was purchased from Difco. Pertussis toxin (BML-G100-0050) was purchased from Enzo. 5Z-7-oxozeaenol (3604) was purchased from Tocris and dissolved in DMSO. Phospho-TAK1 antibody (Ser192; sc-130219), TAK1 antibody (H-5; sc-166562), and NF-κB antibody (sc-8008) were purchased from Santa Cruz Biotechnology. IκBα antibody (L35A5) was purchased from Cell Signaling Technology. Ionized calcium-binding adaptor molecule 1 (Iba-1) antibody (019-19741) used for immunohistochemistry was purchased from WAKO. Iba-1 antibody (ab178847) used for Western blotting was purchased from Abcam. Phospho-p38MAPK (Thr180) antibody (E1A3457), p38MAPK antibody (E1A6456), phospho-JNK1/2/3 (Thr183 + Tyr185) antibody (E1A3318), JNK1/2/3 antibody (E1A6318), phospho-c-Jun (Ser63) antibody (E1A0393-2), c-Jun antibody (E1A6090), phospho-ERK1/2 (Phospho-Y204) antibody (E1A1014), ERK1/2 antibody (E1A0155), and GAPDH (E1A7021) antibody were purchased from EnoGene (Nanjing, China). Actin antibody, HRP-conjugated goat anti-mouse, HRP-conjugated goat anti-rabbit antibody, BeyoECL Plus, Enhanced BCA Protein Assay Kits, and RIPA Lysis Buffer and PMSF for Western blotting were purchased from Beyotime (Shanghai, China). Protease phosphatase inhibitor cocktail (1861281) was purchased from Thermo Fisher Scientific.

### EAE Induction

For delivery of 5Z-7-oxozeaenol, 8- to 9-week-old female mice were subjected to lateral ventricle puncture and catheterized with tubes at 1 week before induction. To induce EAE, 9- to 10-week-old female mice were subcutaneously injected in the groin and axilla with 200 μg MOG_35-55_ in phosphate-buffered saline (PBS) emulsified in an equal volume of complete Freund’s adjuvant (CFA) containing 0.5 mg of *M. tuberculosis* H37RA. As a control, mice were immunized with PBS emulsified in an equal volume of CFA containing same amount of H37RA. All mice were intraperitoneally injected with 400 ng pertussis toxin at the time of immunization and 48 h later.

### Neurological Deficit Evaluation

Mice were weighed and scored blindly by a trained observer every day starting at the day after immunization as follows (Goldmann et al., 2013): 0, no detectable symptoms of EAE; 0.5 distal paralyzed tail; 1.0, completely paralyzed tail; 1.5, paralyzed tail and hind limb weakness; 2, unilateral partial hind limb paralysis; 2.5, bilateral partial hind limb paralysis; 3, complete bilateral hind limb paralysis; 3.5, complete hind limb paralysis and unilateral forelimb paralysis; 4, total paralysis of fore- and hind limbs.

### 5Z-7-Oxozeaenol Dose Screening and Treatment Protocol

To identify the effective dose of 5Z-7-oxozeaenol for treating EAE, we assessed the ability of 0.8, 1.6, and 3.2 μg 5Z-7-oxozeaenol to cure EAE. Mice were randomly divided into four groups: (1) DMSO-EAE group: mice were given 2 μl DMSO by intracerebroventricular administration every 3 days from the first day of immunization to day 21 after the immunization; (2) 5Z-7-oxozeaenol 0.8 μg-EAE group, mice were given 0.8 μg 5Z-7-oxozeaenol in 2 μl DMSO; (3) 5Z-7-oxozeaenol 1.6 μg-EAE group, mice were given 1.6 μg 5Z-7-oxozeaenol in 2 μl DMSO; (4) 5Z-7-oxozeaenol 3.2 μg-EAE group, mice were given 3.2 μg 5Z-7-oxozeaenol in 2 μl DMSO. Each group had four mice. Mice were scored blindly by a trained observer every day starting at the day after immunization to the end of the experiment. All mice were sacrificed at day 21 after immunization. The results showed that 5Z-7-oxozeaenol 1.6 μg exerted a protective effect on EAE from day 19 after immunization. Therefore, we used 1.6 μg 5Z-7-oxozeaenol for the remainder of the study.

To evaluate the therapeutic time-window of 5Z-7-oxozeaenol (1.6 μg/2 μl) for EAE, mice were randomly divided into five groups according to the immunizing inducer (CFA or MOG_35-55_) and 5Z-7-oxozeaenol treatment schedule. (1) DMSO-CFA (negative control) group: mice were immunized with PBS and given 2 μl DMSO by intracerebroventricular administration every 3 days from the first day of immunization to the termination of the experiment. (2) DMSO-EAE (model control) group: mice were immunized with MOG_35-55_ instead of PBS and given DMSO as in the DMSO-CFA group. (3) 5Z-7-oxozeaenol 1.6 μg (0→12 d)-EAE (induction phase-treatment) group: mice were immunized with MOG_35-55_ and given 5Z-7-oxozeaenol (1.6 μg/2 μl) every 3 days from the first day of immunization to day 12 after immunization. (4) 5Z-7-oxozeaenol 1.6 μg (12→21 d)-EAE (effector phase-treatment) group: mice were immunized with MOG_35-55_ and given 5Z-7-oxozeaenol (1.6 μg/2 μl) every 3 days from day 12 of immunization to day 21 after immunization. (5) 5Z-7-oxozeaenol 1.6 μg (0→21 d)-EAE (entire phase-treatment) group: mice were immunized with MOG_35-55_ and given 5Z-7-oxozeaenol (1.6 μg/2 μl) every 3 days from the first day of immunization to the termination of the experiment. Every group had nine mice except for the 5Z-7-oxozeaenol 1.6 μg (0→12 d)-EAE group, which had eight mice. All mice were sacrificed at day 21 after immunization.

### Histology and Immunohistochemistry

Mice were perfused with 4% paraformaldehyde in 0.1 M phosphate buffer on day 21 after immunization. The spinal cords were removed, post-fixed in the same fixative and paraffin embedded. Four-micrometer-thick paraffin-embedded sections of lumbar segment of the spinal cord were stained with hematoxylin and eosin (H&E), Luxol fast blue (LFB) and immunohistochemistry to visualize leukocyte infiltration, demyelination, or microglia activation. Iba-1, a marker of microglia, was used to detect microglia activation in the spinal cord.

H&E staining was scored blindly by two independent observers using a semi-quantitative scoring system (Okuda et al., 1999; Xiao et al., 2015) as follows: 0, no inflammation; 1, cellular infiltrate only in the perivascular areas and meninges; 2, mild cellular infiltrate in parenchyma (1–10 cells/section); 3, moderate cellular infiltrate in parenchyma (11–100 cells/section); 4, severe cellular infiltrate in parenchyma (>100 cells/section). LFB staining was also scored by two observers in a blind fashion as follows: 0, no demyelination; 1, mild demyelination; 2, moderate demyelination; 3, severe demyelination (Zhang et al., 2003; Zhen et al., 2015).

The images were observed and photographed under a DM4000 + LED Leica fluorescent microscope. For quantification, five randomly chosen fields from each section were evaluated. Cells were counted in designated areas using Image-J.

### T Cell Recall Assay and ELISA

On day 21 after immunization, splenocytes were mechanically dissociated into single cell suspensions. To prepare a homogeneous cell suspension, dispersed cells were passed through a 70 μm nylon sieve and centrifuged for 5 min at 1000 rpm. The cell pellet was incubated with 5 ml red blood cell lysis buffer for 5 min, and then 10 ml complete media was added to stop the reaction and the lysate was centrifuged for 5 min at 1000 rpm. Splenic cells (2.5 × 10^6^/ml) were plated in 24-well culture plates in RPMI 1640 (Gibco, Grand Island, NY, United States) supplemented with 10% FBS (Gibco), penicillin–streptomycin solution (penicillin 100 U/ml, streptomycin 100 μg/ml; Beyotime Biotechnology, China) and 20 μg/ml MOG_35-55_ were added to the suspension. Splenocytes were cultured at 37°C in a humidified atmosphere of 5% CO_2_. Supernatants were harvested after 48 h, and IL-17A, IFN-γ, TNF-α, and IL-6 concentrations were determined by ELISA (Biolegend, San Diego, CA, United States) according to the manufacturer’s instructions.

### Spinal Cord Cytokine Measurement and ELISA

Mouse spinal cord tissues were homogenized in RIPA buffer containing protease inhibitors and centrifuged at 14,000 rpm for 15 min. The protein in the supernatants was quantified by the BCA method. The supernatant was also used to measure the levels of IL-17A, IFN-γ, TNF-α, and IL-6 by the sandwich ELISA method (Biolegend, San Diego, CA, United States) according to the manufacturer’s instructions. The concentrations of cytokines were expressed as picograms per 100 μg total protein (pg/100 μg total protein).

### Cell Culture and Treatment

The mouse microglia cell line BV2 was cultured in DMEM supplemented with 6% FBS, 100 U/ml penicillin and 100 μg/ml streptomycin at 37°C in humidified atmosphere of 5% CO_2_. After 24 h, the medium was exchanged with DMEM containing 5Z-7-oxozeaenol (500 nM/ml) or DMSO (5 μl/ml). After 30 min, the medium was exchanged again with DMEM with or without LPS (1.0 μg/ml), and the cells were incubated for 6 h. Subsequently, the total protein of BV2 cells was extracted. The expression of TAK1, NF-κB, and MAPK-related proteins were detected by Western blotting.

### Western Blot Analysis

Tissues from the lumbar segment of mouse spinal cords and BV2 cells were lysed in RIPA buffer supplemented with PMSF and protease inhibitor cocktail. Aliquots with equal amounts of proteins (70 μg per lane) were boiled for 5 min, loaded onto a 12% Tris-SDS-polyacrylamide gel (PAGE) except for Iba-1 protein, which loaded onto a 10% Tricine-SDS-PAGE and transferred to PVDF membranes (Millipore). After gel electrophoresis, the membranes were incubated with primary antibodies against Iba-1 (1:1000), phospho-TAK1 (1:200), TAK1 (1:200), NF-κB (1:200), IκBα (1:1000), phospho-p38 (1:1000), p38 (1:1000), phospho-c-Jun (1:1000), c-Jun (1:1000), phospho-ERK1/2 (1:1000), ERK1/2 (1:1000), β-actin (1:1000), or GAPDH (1:1000). After washing, the blots were incubated with horseradish peroxidase-labeled secondary goat anti-rabbit IgG (1:1000) or goat anti-mouse IgG (1:1000) antibody followed by chemiluminescence (ECL) reagents. The protein bands were analyzed using Image-J software.

### Statistical Analyses

Statistical relevance was analyzed by SPSS 20.0 software. All data were presented as mean ± SEM. Daily mean clinical EAE scores among different time points and maximum clinical and histopathological scores between groups were assessed by the Kruskal–Wallis test followed by the Mann–Whitney *U*-test. For other analyses with a single treatment factor, including immunohistochemistry, ELISA, and Western blot analysis, data were analyzed by one-way ANOVA. Comparisons between multiple groups were followed by least a significant difference *post hoc* comparison. GraphPad Prism software was utilized to plot the data, and *P* ≤ 0.05 was considered statistically different.

## Results

### Delivery of 5Z-7-Oxozeaenol 1.6 μg Exerts Protect Effect on EAE

**FIGURE 1:**
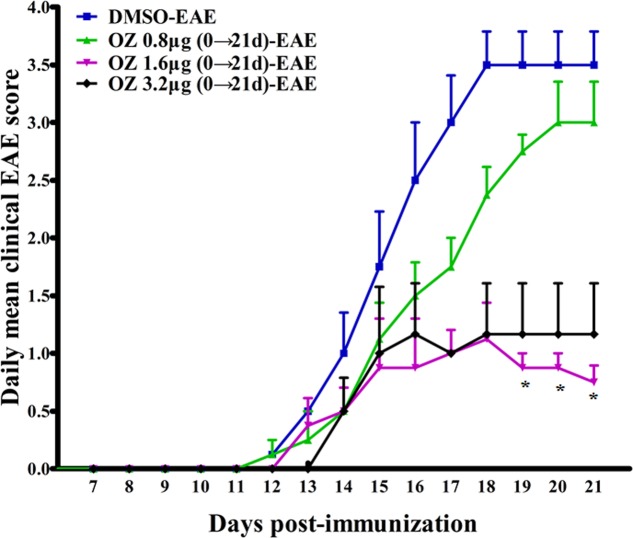
The effect of different doses of 5Z-7-oxozeaenol treatment on the neurological deficit scores in EAE mice. Three doses of 5Z-7-oxozeaenol were used to treat EAE mice. Clinical scores of EAE were recorded from the day of immunization until 21 days after immunization. Each group included four mice. One mouse from the 5Z-7-oxozeaenol 3.2 μg-EAE group died during the experiment. Data are means ± SEM. ^∗^Adjusted *P* < 0.017 versus DMSO-EAE group (Mann–Whitney *U*-test). OZ, 5Z-7-oxozeaenol.

To screen for the effective dose of 5Z-7-oxozeaenol for treating EAE, we administered three dosages of 5Z-7-oxozeaenol to EAE mice. When compared to the DMSO-EAE group, 0.8 μg 5Z-7-oxozeaenol had no protective effect on EAE (*P* > 0.05), and 1.6 and 3.2 μg 5Z-7-oxozeaenol protected mice from EAE from 19 days onward after immunization. However, one mouse in the 5Z-7-oxozeaenol 3.2 μg group died. Therefore, we used 1.6 μg 5Z-7-oxozeaenol for the remainder of the study (**Figure 1**).

### Delivery of 5Z-7-Oxozeaenol during the Effector Phase (12–21 d) and the Entire Phase (0–21 d) Alleviates the Severity of EAE

**FIGURE 2:**
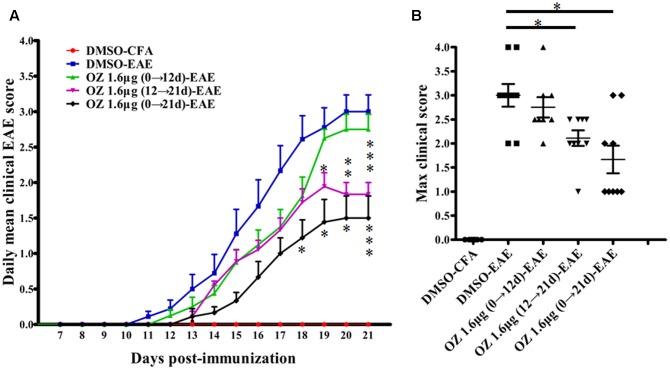
The effect of 5Z-7-oxozeaenol treatment on the neurological deficit scores in EAE mice. The EAE mouse model was established by injection of MOG_35-55_. Clinical scores of EAE were recorded from the day of immunization until 21 days after immunization. **(A)** Daily mean clinical score of EAE in five groups. **(B)** Max clinical score of EAE in five groups. 5Z-7-oxozeaenol 1.6 μg (0→12 d)-EAE group, *n* = 8; the other groups, *n* = 9. Data are means ± SEM. ^∗^Adjusted *P* < 0.017, ^∗∗^*P* < 0.01 and ^∗∗∗^*P* < 0.001 versus DMSO-EAE group (Mann–Whitney *U*-test). OZ, 5Z-7-oxozeaenol.

To evaluate the efficacy of TAK1 inhibition in treating EAE mice, we applied 5Z-7-oxozeaenol at different phases and used clinical scoring to assess neurological deficits over a period of 21 days after EAE induction with MOG_35-55_. The DMSO-CFA (negative control) group showed no visible neurobehavioral deficits during the entire period. In the DMSO-EAE (model control) group, neurological deficits began to appear on the 11th day, worsened gradually and reached a peak on the 18th day. Therefore, we used day 12 as a cutoff point for application of 5Z-7-oxozeaenol either prior to (0→12 d; induction phase), subsequent to (12→21 d; effector phase), or both prior and subsequent to (0→21 d; entire phase) disease onset. Compare to the DMSO-EAE group, both the 5Z-7-oxozeaenol 1.6 μg (12→21 d)-EAE and 5Z-7-oxozeaenol 1.6 μg (0→21 d)-EAE groups showed efficiently improved clinical outcomes from 18/19 day post-immunization (d.p.i), as assessed by the daily mean clinical EAE scores (**Figure 2A**). Both of these groups also had lower max clinical scores, which indicates that mice in these groups had attenuated symptoms (**Figure 2B**). However, the 5Z-7-oxozeaenol 1.6 μg (0→12 d)-EAE group showed no significant effect on the daily mean clinical EAE scores and max clinical scores relative to the scores of the DMSO-EAE group (**Figure 2**). These results indicate that 5Z-7-oxozeaenol effectively attenuates the symptoms of EAE when applied during the effector phase or entire phase.

### Delivery of 5Z-7-Oxozeaenol during the Effector Phase (12–21 d) and Entire Phase (0–21 d) Inhibits Inflammation and Demyelination in the Spinal Cords of EAE Mice

**FIGURE 3:**
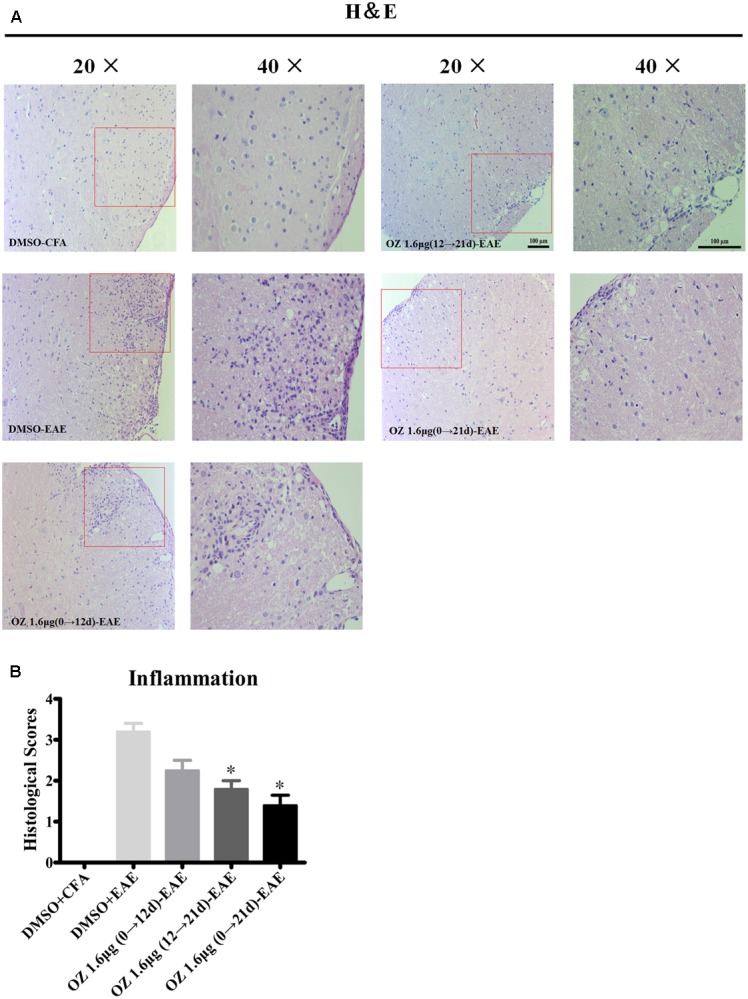
The effect of 5Z-7-oxozeaenol treatment on lymphocyte infiltration of spinal cords from EAE mice. H&E staining was used to evaluate inflammation in the lumbar intumescence spinal cord in each group at 21 days post-immunization with MOG_35-55_. Scores were determined by a semi-quantitative method. **(A)** Representative H&E staining of spinal cord sections. **(B)** Quantification of inflammation in the spinal cord. Each group included five mice, except for the 5Z-7-oxozeaenol 1.6 μg (0→12 d)-EAE group, which only had four mice because one died. ^∗^Adjusted *P* < 0.017 versus DMSO-EAE group (Mann–Whitney *U*-test). Data are means ± SEM. OZ, 5Z-7-oxozeaenol. Scale bar = 100 μm.

To elucidate the basis for the beneficial effect of 5Z-7-oxozeaenol, we further examined the pathological changes in EAE mice upon 5Z-7-oxozeaenol treatment. On the 21^st^ day after immunization, we performed H&E staining to examine inflammatory cell infiltration. Consistent with the clinical findings, inflammatory infiltration was not apparent in the lumbar segment of the spinal cord for the DMSO-CFA group, but was greatly elevated in the parenchyma of the DMSO-EAE group. The infiltration was reduced in the 5Z-7-oxozeaenol 1.6 μg (12→21 d)-EAE and 5Z-7-oxozeaenol 1.6 μg (0→21 d)-EAE groups, for which inflammatory cell infiltration only was apparent in the perivascular areas and meninges (**Figure 3**). However, the infiltration in the 5Z-7-oxozeaenol 1.6 μg (0→12 d)-EAE group was not statistically reduced.

**FIGURE 4:**
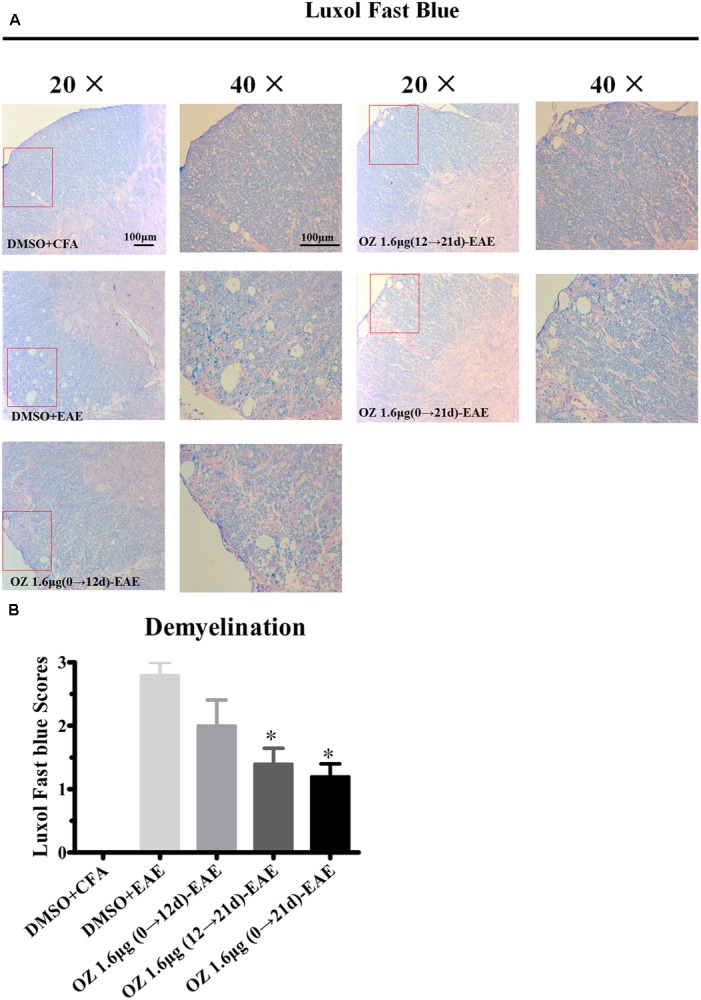
The effect of 5Z-7-oxozeaenol treatment on demyelination of the spinal cord in EAE mice. LFB staining was used to evaluate the demyelination of lumbar intumescence spinal cord in each group at 21 days post-immunization with MOG_35-55_. Staining scores were determined by a semi-quantitative method. **(A)** Representative LFB staining of spinal cord sections. **(B)** Quantification of demyelination in the spinal cord. Each group included five mice, except for the 5Z-7-oxozeaenol 1.6 μg (0→12 d)-EAE group, which only had four mice because one died. ^∗^Adjusted *P* < 0.017 versus DMSO-EAE group (Mann–Whitney *U*-test). Data are means ± SEM. OZ, 5Z-7-oxozeaenol. Scale bar = 100 μm.

To evaluate the effects of 5Z-7-oxozeaenol on demyelination, we also performed LFB staining. A similar pattern was observed of statistical reduction in demyelination levels in EAE mice upon treatment with 5Z-7-oxozeaenol on days 12→21 and days 0→21, but not days 0→12 (**Figure 4**). Therefore, these results suggest that administration of 5Z-7-oxozeaenol during the effector phase and entire phase effectively reduces EAE severity by suppressing spinal cord inflammation and demyelination.

### Delivery of 5Z-7-Oxozeaenol during the Effector Phase (12–21 d) and the Entire Phase (0–21 d) Decreases the Secretion Pro-inflammatory Cytokines in Splenocytes and the CNS

**FIGURE 5:**
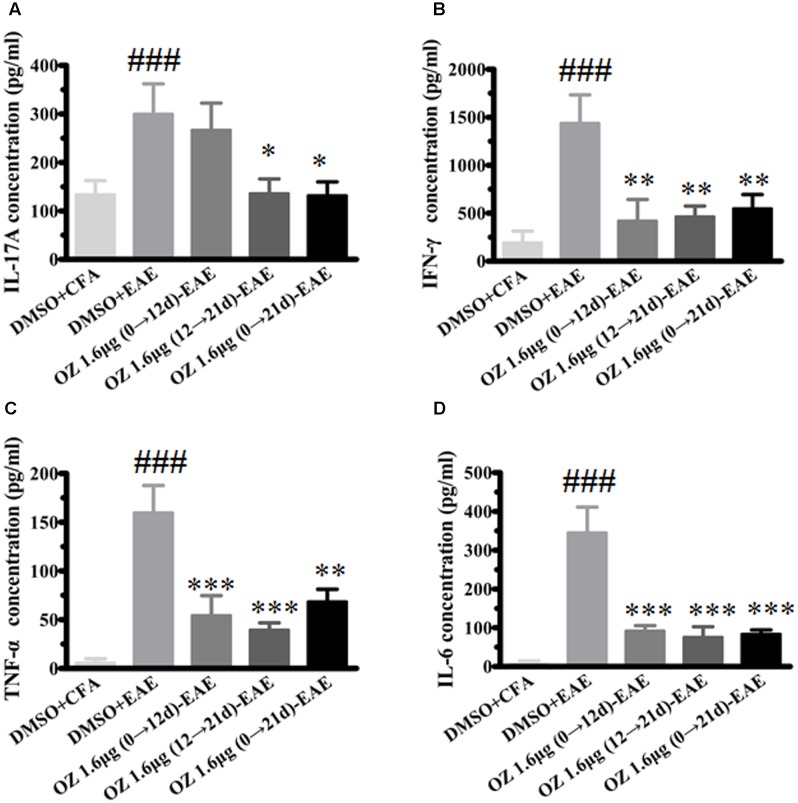
The effects of 5Z-7-oxozeaenol treatment on pro-inflammatory cytokines produced by splenocytes in the EAE model. Splenocytes were harvested and stimulated with 20 μg MOG_35-55_ for 48 h. The supernatants were harvested and analyzed for cytokine content by ELISA. **(A)** Quantification of IL-17A levels. **(B)** Quantification of IFN-γ levels. **(C)** Quantification of TNF-α levels. **(D)** Quantification of IL-6 levels. The data are expressed as mean ± SEM (*n* = 4 mice per group). ^∗^*P* < 0.05, ^∗∗^*P* < 0.01, ^∗∗∗^*P* < 0.001 versus DMSO-EAE group, ^###^*P* < 0.001 versus DMSO-CFA group (one-way ANOVA). OZ, 5Z-7-oxozeaenol.

**FIGURE 6:**
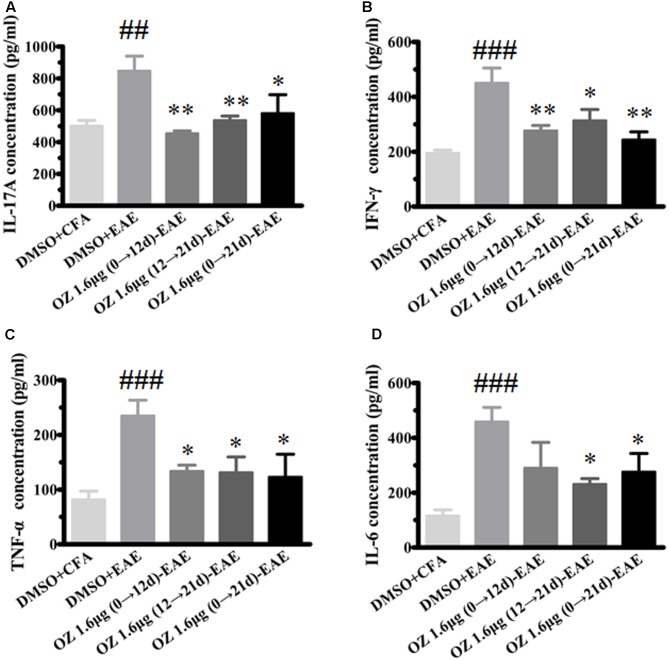
The effects of 5Z-7-oxozeaenol treatment on pro-inflammatory cytokines produced in the CNS in the EAE model. Spinal cords were homogenized. The supernatant of protein were quantify by BCA method. The supernatants were analyzed for cytokine content by ELISA. **(A)** Quantification of IL-17A levels. **(B)** Quantification of IFN-γ levels. **(C)** Quantification of TNF-α levels. **(D)** Quantification of IL-6 levels. The data are expressed as mean ± SEM (*n* = 4 mice per group). ^∗^*P* < 0.05, ^∗∗^*P* < 0.01 versus DMSO-EAE group, ^##^*P* < 0.01, ^###^*P* < 0.001 versus DMSO-CFA group (one-way ANOVA). OZ, 5Z-7-oxozeaenol.

In CNS inflammatory diseases, pro-inflammatory cytokines are primarily produced by tissue-invading immune cells. When delivered to the CNS, they are the dominant forces that orchestrate the ensuing inflammatory cascade. These cytokines can act on CNS-resident cells (microglia, astrocytes) to elicit the production of a more diverse array of cytokines, which in turn not only helps to recruit more leukocytes, but also influences the behavior of the tissue-invading cells themselves, thus fueling the inflammatory cascade (Becher et al., 2016). To dissect the potential anti-inflammatory mechanisms of 5Z-7-oxozeaenol in EAE mice, we first performed T cell recall response assays. Then, we detected the pro-inflammatory cytokines in the CNS. We measured the pro-inflammatory cytokine expression in culture supernatants of splenocytes after MOG_35-55_ re-stimulation and the CNS from CFA/MOG_35-55_ immunized mice by ELISA. Compared to the levels in the DMSO-CFA group, the DMSO-EAE group showed elevated IL-17A, IFN-γ, TNF-α, and IL-6 in splenocytes (**Figure 5**) and in the CNS (**Figure 6**). In the spleen, all of the 5Z-7-oxozeaenol-treated groups displayed significantly decreased levels of IFN-γ, TNF-α, and IL-6 (**Figures 5B–D**), whereas the 5Z-7-oxozeaenol 1.6 μg (12→21 d)-EAE and 5Z-7-oxozeaenol 1.6 μg (0→21 d)-EAE groups displayed decreased IL-17A levels (**Figure 5A**). In the CNS, all of the 5Z-7-oxozeaenol-treated groups displayed significantly decreased levels of IL-17A, IFN-γ, TNF-α (**Figures 6A–C**), whereas the 5Z-7-oxozeaenol 1.6 μg (12→21 d)-EAE and 5Z-7-oxozeaenol 1.6 μg (0→21 d)-EAE groups displayed decreased IL-6 levels (**Figure 6D**). These results suggest that 5Z-7-oxozeaenol has a potent anti-inflammatory function in the CNS and spleen, regardless of administration in the induction and/or effector phase.

### Delivery of 5Z-7-Oxozeaenol during the Effector Phase (12–21 d) and the Entire Phase (0–21 d) Is Associated with Decreased Microglia Activation in the Spinal Cord

**FIGURE 7:**
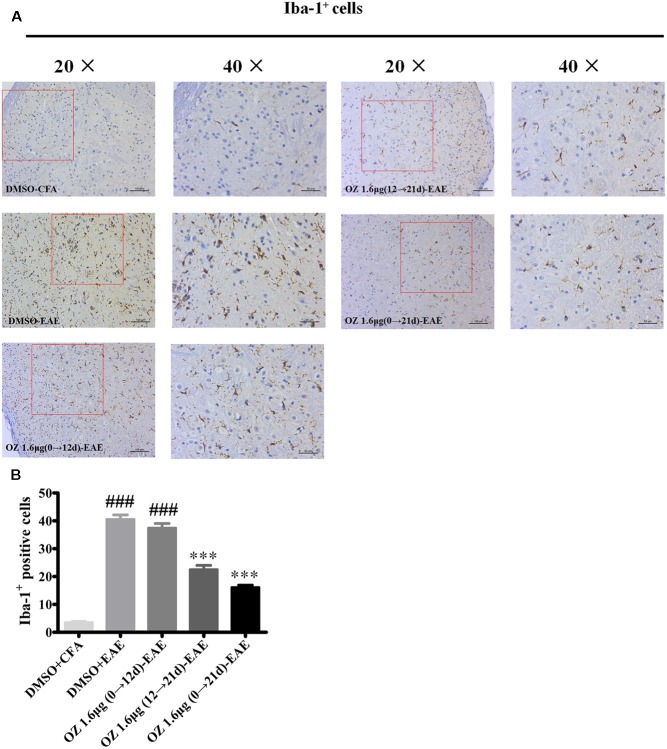
The effect of 5Z-7-oxozeaenol treatment on Iba-1-positive microglia in the spinal cords of EAE mice. Immunohistochemistry was used to evaluate the Iba-1-positive microglia in the lumbar intumescence spinal cord in each group. Counts of Iba-1-positive microglia were determined by quantitative method. Five fields from each mouse were analyzed. **(A)** Representative immunohistochemistry of Iba-1-positive microglia. **(B)** Quantification of Iba-1-positive microglia in the spinal cord. Each group included five mice, except for the 5Z-7-oxozeaenol 1.6 μg (0→12 d)-EAE group, which only had four mice because one died. ^∗∗∗^*P* < 0.001 versus DMSO-EAE group, ^###^*P* < 0.001 versus DMSO-CFA group (one-way ANOVA). Data are means ± SEM. OZ, 5Z-7-oxozeaenol. Scale bar = 50 μm for 20×, 100 μm for 40×.

Microglia serve as one of the most important immunocompetent cells and a first line of defense in the CNS in response to tissue damage. Excessive or continuous activation of microglia leads to direct neurotoxicity, characterized by the production of pro-inflammatory cytokines (such as TNF-α, IL-6, and NO) and impaired neural cell function (Hanisch, 2002; Xu et al., 2016). Secreted cytokines and other harmful factors will promote the occurrence and development of inflammatory reaction. We wondered whether the neuroprotective effects of 5Z-7-oxozeaenol in the EAE mouse model might be explained by 5Z-7-oxozeaenol-induced inhibition of microglia activation. To assess this possibility, lumbar segments of spinal cord cross sections from CFA/EAE mice were subjected to immunohistochemistry for Iba-1, a marker for microglia activation. Few Iba-1-positive microglia were observed in the DMSO-CFA group, but a large number of Iba-1-positive microglia with more irregular cell body hypertrophy and shorten processes (active microglia) were observed in the DMSO-EAE group. The morphology and Iba-1-positivity in the 5Z-7-oxozeaenol 1.6 μg (0→12 d)-EAE group were similar to that of the DMSO-EAE group, but the 5Z-7-oxozeaenol 1.6 μg (12→21 d)-EAE and 5Z-7-oxozeaenol 1.6 μg (0→21 d)-EAE groups displayed suppressed levels of microglia activation with improved morphology (**Figure 7**).

**FIGURE 8:**
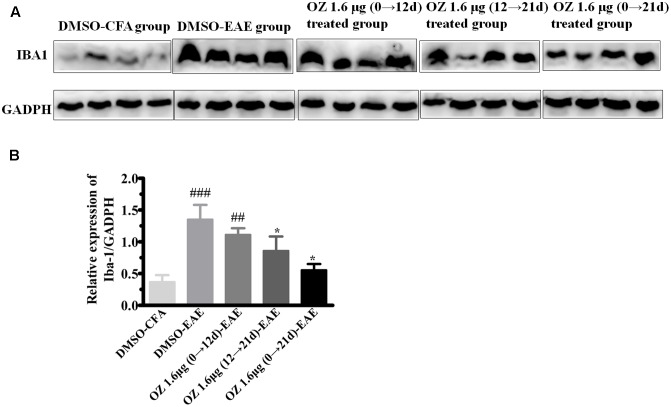
Western blotting analysis of Iba-1 protein in spinal cord after 5Z-7-oxozeaenol treatment of EAE mice. The total protein expression of Iba-1, a marker of microglia, was analyzed by Western blotting at 21 days post-immunization. **(A)** Immunodensity of Iba-1 levels. **(B)** Quantification of Iba-1 levels. The data are expressed as mean ± SEM (*n* = 4 mice per group). ^∗^*P* < 0.05 versus DMSO-EAE group, ^##^*P* < 0.01, ^###^*P* < 0.001 versus DMSO-CFA group (one-way ANOVA). OZ, 5Z-7-oxozeaenol.

To verify the differences in the total number of activated microglia between groups we performed Western blotting. Consistent with the immunohistochemistry results mentioned above, the DMSO-EAE group mice showed an increased immunodensity of Iba-1. The 5Z-7-oxozeaenol 1.6 μg (0→12 d)-EAE group also had a high level of Iba-1 immunodensity (*P* > 0.05 compared to the DMSO-EAE group); however, the 5Z-7-oxozeaenol 1.6 μg (12→21 d)-EAE and 5Z-7-oxozeaenol 1.6 μg (0→21 d)-EAE groups had reduced Iba-1 immunodensity (*P* < 0.05) (**Figure 8**). Thus, the improved clinical outcome of 5Z-7-oxozeaenol administration to EAE mice may be explained by a dampened inflammatory immune response that results from reduced microglia activation (**Figure 8**).

### Application of 5Z-7-Oxozeanol during the Effector Phase (12–21 d) and the Entire Phase (0–21 d) Reduces the Level of Activated TAK1 in the EAE Model

**FIGURE 9:**
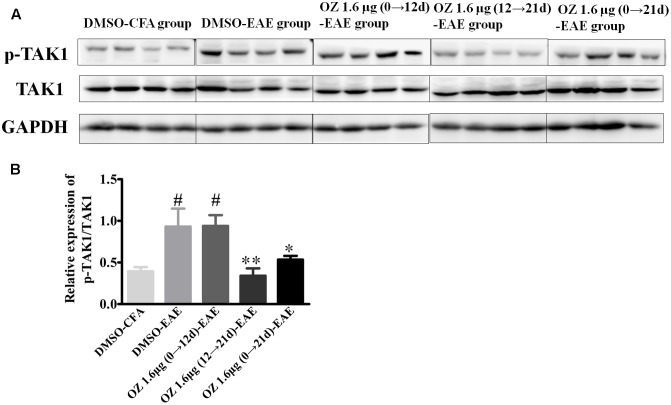
The effects of 5Z-7-oxozeaenol treatment on the TAK1 signaling pathway in spinal cords in the EAE model. The total protein expression of TAK1 was analyzed by Western blotting of spinal cords from EAE mice at 21 days post-immunization. **(A)** Immunodensity of p-TAK1 and TAK1 levels. **(B)** Quantification of p-TAK1/TAK1 levels. The data are expressed as mean ± SEM (*n* = 4 mice per group). ^∗^*P* < 0.05, ^∗∗^*P* < 0.01 versus DMSO-EAE group, ^#^*P* < 0.05 versus DMSO-CFA group (one-way ANOVA). OZ, 5Z-7-oxozeaenol.

5Z-7-oxozeaenol is known as an inhibitor that potently suppresses TAK1 activation. To confirm the influence of 5Z-7-oxozeaenol administration on TAK1 in the setting of EAE, we analyzed spinal cord homogenates by Western blotting of phosphorylated (activated) TAK1 and total TAK1. Compared to the phospho-TAK1 level in the DMSO-CFA group, the level in the DMSO-EAE group was increased. The 5Z-7-oxozeaenol 1.6 μg (12→21 d)-EAE and 5Z-7-oxozeaenol 1.6 μg (0→21 d)-EAE groups displayed reduced phospho-TAK1 levels compared to the level in the DMSO-EAE group, whereas the level in the 5Z-7-oxozeaenol 1.6 μg (0→12 d)-EAE group was not significantly different (**Figure 9**). These finding verify the function of 5Z-7-oxozeaenol in suppressing TAK1 activation and support the role of TAK1 in mediating the neurodegenerative outcome of EAE.

### Delivery of 5Z-7-Oxozeanol during the Effector Phase (12–21 d) and the Entire Phase (0–21 d) Inhibits p38MAPK, JNK, and ERK Signaling, but not NF-κB Signaling

**FIGURE 10:**
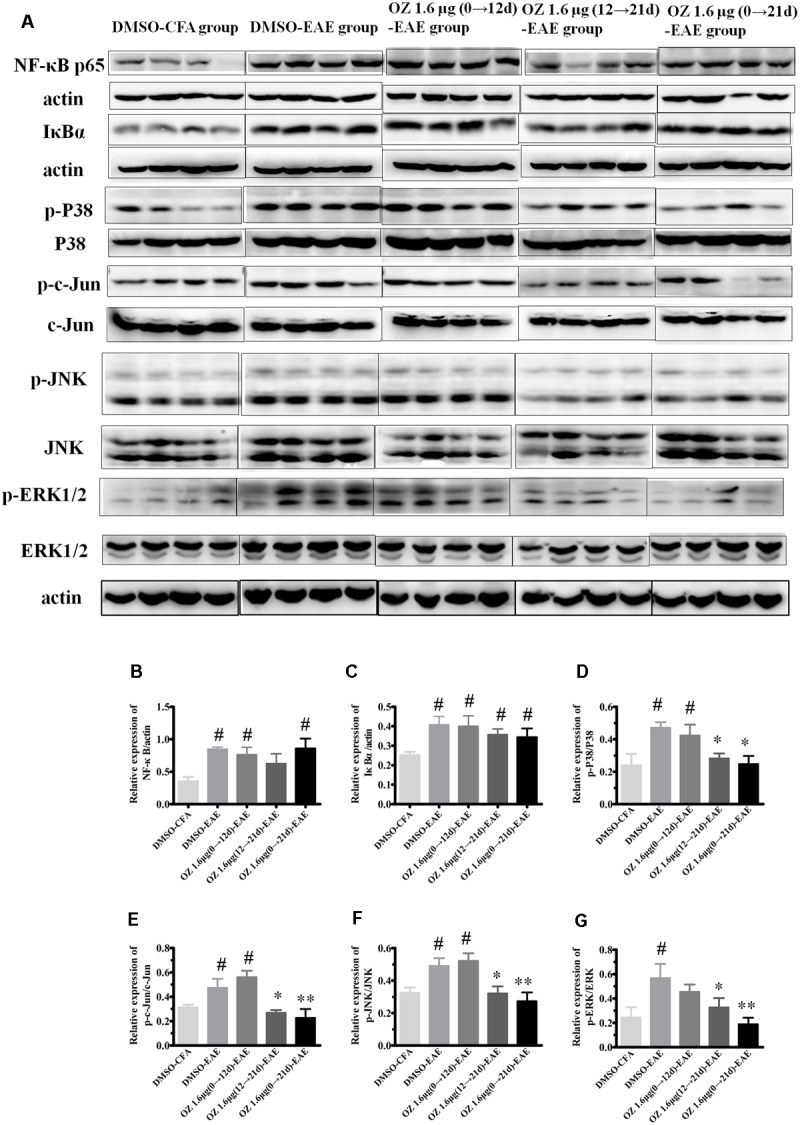
The effects of 5Z-7-oxozeaenol on NF-κB, p38MAPK, JNK, and ERK signaling pathways in spinal cords from EAE mice. Lumbar segments of spinal cords from EAE mice were harvested at 21 days post-immunization. The total protein expression of NF-κB, IκBα, p-p38, p38, p-c-Jun, c-Jun, p-JNK, JNK, p-ERK1/2, ERK1/2 was analyzed by Western blot. **(A)** Immunodensity of NF-κB, IκBα, p-p38, p38, p-c-Jun, c-Jun, p-ERK, and ERK levels. **(B–G)** Quantification of NF-κB, IκBα, p-p38, p38, p-c-Jun, c-Jun, p-JNK, JNK, p-ERK, and ERK levels. The data are expressed as mean ± SEM (*n* = 4 mice per group). ^∗^*P* < 0.05, ^∗∗^*P* < 0.01 versus DMSO-EAE group, ^#^*P* < 0.05 versus DMSO-CFA group (one-way ANOVA). OZ, 5Z-7-oxozeaenol.

TAK1 is a common upstream activator of NF-κB, p38MAPK, JNK, and ERK signaling pathways. To determine the effect of 5Z-7-oxozeaenol on these downstream signaling cascades in the EAE mouse, we performed Western blotting of spinal cord homogenates from DMSO/5Z-7-oxozeaenol treated CFA/EAE mice (**Figure 10**). The expression of NF-κB and its inhibitor IκBα was activated by the induction of EAE; however, 5Z-7-oxozeaenol did not suppress the activation of the NF-κB pathway under any of the conditions of 5Z-7-oxozeanol administration. The substantial levels of IκBα in the presence of 5Z-7-oxozeaenol were barely changed, indicative of restricted activity of NF-κB signaling in the spinal cord (**Figures 10A–C**). However, the p38MAPK pathway was activated by EAE induction, and the activation was attenuated in the 5Z-7-oxozeaenol 1.6 μg (12→21 d)-EAE and 5Z-7-oxozeaenol 1.6 μg (0→21 d)-EAE groups, as evidenced by the phosphorylation of p38MAPK in these groups compared to DMSO-EAE group. However, no statistical reduction in p38MAPK phosphorylation was observed for the 5Z-7-oxozeaenol 1.6 μg (0→12 d)-EAE group (**Figures 10A,D**). Similarly, the phosphorylation of c-Jun, a downstream signal molecule of JNK, and the phosphorylation of JNK were increased after EAE induction, and these increases were attenuated in the 5Z-7-oxozeaenol 1.6 μg (12→21 d)-EAE and 5Z-7-oxozeaenol 1.6 μg (0→21 d)-EAE groups (**Figures 10A,E,F**). The phospho-ERK1/2 levels were also altered after EAE induction, and the application of 5Z-7-oxozeaenol in the effector (12→21 d) and entire (0→21 d) phase reduced ERK1/2 activation (**Figures 10A,G**). According to these results, we conclude that administration of 5Z-7-oxozeaenol inhibits the p38MAPK, JNK and ERK pathways, all of which may play important roles in mediating its effects in inactivating microglia and reducing neuro-inflammation.

### Administration of 5Z-7-Oxozeaenol Interferes with LPS-Induced MAPK Signaling in BV2 Cells

**FIGURE 11:**
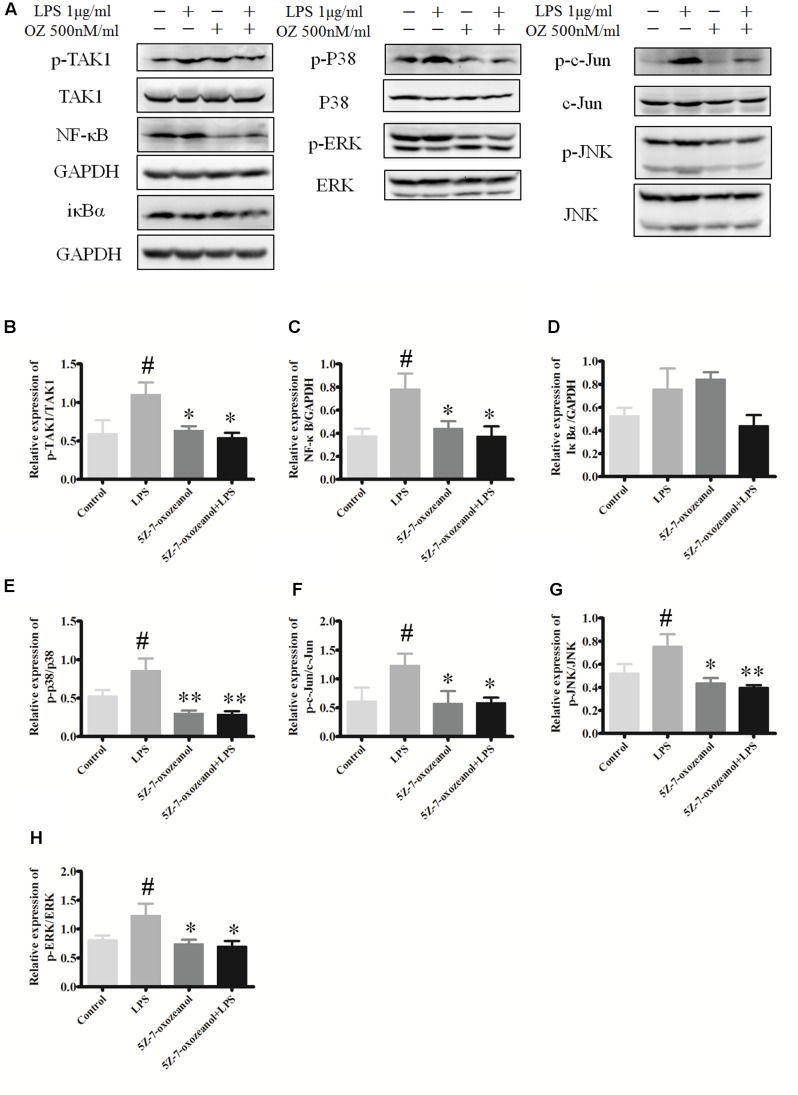
5Z-7-oxozeaenol inhibits LPS-induced TAK1, NF-κB, and MAPK activation in BV2 cells. Experiments were divided into four groups as follows: control group, BV2 cells treated with DMEM complete medium; LPS group, BV2 cell treated with LPS (1.0 μg/ml) for 6 h; 5Z-7-oxozeaenol group, BV2 cells pre-treated with 5Z-7-oxozeaenol (500 nM) for 30 min and then washed with PBS twice, followed by DMEM complete medium treatment for 6 h; 5Z-7-oxozeaenol + LPS group, BV2 cells pre-treated with 5Z-7-oxozeaenol (500 nM) for 30 min and then washed with PBS twice, followed by LPS (1.0 μg/ml) stimulation for 6 h. Expression was evaluated by Western blotting. **(A)** Immunodensity of TAK1, NF-κB, and MAPK levels. **(B–H)** Quantification of proteins level. Experiments were repeated three times, and similar results were obtained. The data are expressed as mean ± SEM ^∗^*P* < 0.05, ^∗∗^*P* < 0.01 versus LPS group. ^#^*P* < 0.05 versus control group (one-way ANOVA). OZ, 5Z-7-oxozeaenol.

To support the possibility that the *in vivo* effects of 5Z-7-oxozeaenol on MAPK signaling may be mediated by direct effects on microglia, we examined the *in vitro* the ability of 5Z-7-oxozeaenol to alter MAPK signaling pathways in microglia. The mouse microglia cell line BV2 was pre-treated with DMSO or 500 nM 5Z-7-oxozeaenol for 30 min prior to activation of MAPK signaling by treatment with LPS (1 μg/ml). LPS increased the levels of p-TAK1, NF-κB, p-p38, p-c-Jun p-JNK, and p-ERK1/2. As expected, 5Z-7-oxozeaenol (500 nM) treatment blocked LPS-induced TAK1 phosphorylation at Ser192. 5Z-7-oxozeaenol did not affect the inflammatory NF-κB signaling cascade, as evidenced by the expression levels of its inhibitor IκBα, which were almost the same with and without 5Z-7-oxozeaenol. However, LPS-induced MAPK activation was largely inhibited by 5Z-7-oxozeaenol co-treatment (**Figure 11**). 5Z-7-oxozeaenol led to a reduction in LPS-induced MAPK activation, whereas no effect on NF-κB activation was observed. These data demonstrate that 5Z-7-oxozeaenol has the potential to inhibit MAPK activation in a microglia cell line. Collectively, our data suggest that 5Z-7-oxozeaenol is a potent inhibitor of inflammatory cascades in microglial cells, which may contribute to its neuroprotective effects within the CNS parenchyma.

## Discussion

TAK1 has been found to play a critical role in innate and adaptive immunity, DNA damage response and inflammatory signaling (Dai et al., 2012). However, the role of TAK1 in autoimmune diseases of the brain had not been well characterized until recently, when conditional depletion of TAK1 in microglia was shown to block p65, JNK, ERK1/2, and p38MAPKK to protect mice from EAE, potentially by impeding the infiltration of peripheral immune cells into the CNS and overall dampening of inflammatory immune response. Microglia-specific expression of TAK1 is essential for the pathogenesis of autoimmune inflammation of the CNS (Goldmann et al., 2013). In this context, we examined the effect of TAK1 inhibition with 5Z-7-oxozeaenol in ameliorating the symptoms and cellular and molecular effects of EAE. Consistent with the results of Goldmann, we demonstrated that 5Z-7-oxozeaenol administration attenuates the production of pro-inflammatory cytokines and the activation of microglia by interfering with p38MAPK, JNK, and ERK signaling pathways, whereas it had no effect on NF-κB activation. Here, for the first time, we identified a unique effective therapeutic time-window of 5Z-7-oxozeaenol in MOG-induced EAE. Our results demonstrate that 5Z-7-oxozeanol shows therapeutic efficacy when administered either 12→21 d.p.i., corresponding to the symptomatic stage, or 0→21 d.p.i., corresponding to the entire stage.

Pro-inflammatory cytokines play a prominent role in classical neuroinflammatory diseases, such as MS and encephalitides. We demonstrated that splenocytes extracted from 5Z-7-oxozeaenol-treated EAE mice display a significant decrease in IL-17A, IFN-γ, TNF-α, and IL-6 production in response to MOG re-stimulation. In EAE mice model, the BBB is compromised. Immune cells can invade to the CNS through a compromised BBB and vice versa. 5Z-7-oxozeaenol may cross the BBB to exert its effect on splenocytes. Similar results have been described for other methods of TAK1 inhibition. For example, *in vivo* RNAi-mediated silencing of TAK1 in the mouse significantly decreases IL-17A, IFN-γ, and TNF-α secretion produced by the spleen in collagen-induced arthritis (Courties et al., 2010). 5Z-7-oxozeaenol also inhibits pro-inflammatory cytokines in the CNS. However, there is some disagreement in regards to the therapeutic time-window of 5Z-7-oxozeaenol for ameliorating the neurological deficit score, EAE pathology and pro-inflammatory cytokine production, which cannot be simply explained by limiting T cell responses or pro-inflammatory cytokines. These incongruences are likely to be explained by the contribution of other cells that contribute to the pathogenesis of EAE, such as microglia.

Microglia serve as the first scavengers after CNS insult, and activated microglia are both major producers and targets of pro-inflammatory cytokines (Zhou et al., 2015). Therefore, we also evaluated whether the protection by 5Z-7-oxozeaenol of EAE pathology might due to anti-inflammatory effects on microglia that reside within the CNS. Our results demonstrate that the ameliorated EAE pathologic features and clinical severity after treatment of 5Z-7-oxozeaenol during the effector phase (12→21 d.p.i.) and the entire phase (0→21 d.p.i.), accompanied by a marked reduction in CNS inflammation and demyelination, were coincident with decreased microglia activation in the CNS. This highlights the potent ability of 5Z-7-oxozeaenol to regulate microglia function, which would lead to a suppressed pro-inflammatory milieu in the CNS of EAE mice. Therapeutic approaches designed to modulate the activation of microglia are known to be beneficial to EAE. For example, 18 β-glycyrrhetinic acid suppresses the progression of EAE via inhibition of microglia activation (Zhou et al., 2015). Furthermore, pertussis toxin modulates the microglia and T cell profile to protect EAE (Yin et al., 2014). Finally, cannabidiol decreases spinal microglial activation and ameliorates symptoms of EAE in C57BL/6 mice (Kozela et al., 2011).

TAK1 is highly expressed in the brain (Yamaguchi et al., 1995). However, it is currently unclear whether TAK1 is also activated in the brains of MS patients, while it has been shown that TAK1 and downstream NF-κB and MAPK signaling pathways are activated in EAE mice (Shin et al., 2003). Our results verify that the NF-κB, p38MAPK, JNK, and ERK pathways are activated by EAE induction. Furthermore, 5Z-7-oxozeaenol interfered with the p38MAPK, JNK, and ERK signaling cascades, but not the NF-κB signaling pathway. These results suggest that 5Z-7-oxozeaenol shows a level of selectivity in the down-stream signaling cascades that are inhibited. Similar to these findings, mice treated with 5Z-7-oxozeaenol show a significant reduction in the activity of p38MAPK after MCAO, but have no effect on JNK (Neubert et al., 2011). Though the NF-κB signaling pathway was not blocked by *in vivo* 5Z-7-oxozeaenol administration in our study, blockade of the NF-κB signaling pathway is known to enhance the capacity of immature dendritic cells to induce antigen-specific tolerance in EAE (Iruretagoyena et al., 2006). Furthermore, NF-κB inhibition has been shown to be an effective therapeutic approach for other autoimmune diseases. For example, transgenic inhibition of astroglial NF-κB protects experimental optic neuritis mice from optic nerve damage and retinal ganglion cell loss (Brambilla et al., 2012). Therefore, a synergistic approach could potentially enhance the effectiveness of 5Z-7-oxozeaenol.

However, even in the absence of NF-κB inhibition, the ability of 5Z-7-oxozeaenol to simultaneously inhibit each of the three MAPK pathways could explain its efficacy. In particular, p38MAPK plays a prominent role in multiple inflammatory diseases. Oral application of a highly specific p38 inhibitor, UR-5269, markedly reduces clinical symptoms of EAE (Namiki et al., 2012). Furthermore, inhibition of p38MAPK exhibits protective effects in animal models of rheumatoid arthritis (Brown et al., 2004). Inhibitors of p38MAPK have been the subject of clinical trials for the treatment of rheumatoid arthritis and psoriasis (Cohen, 2009); however, none of the inhibitors have progressed to phase III because of toxicity issues. Such side effects are thought to be due to the inhibition of p38MAPK feedback control loops, resulting in the hyperactivation of TAK1 and JNK kinases (Cohen, 2009). Therefore, TAK1 inhibition has an advantage of simultaneously inhibition of several of the pathways that are susceptible to feedback regulation.

To support the role of 5Z-7-oxozeaenol in microglia, we also assessed its *in vitro* ability to suppress NF-κB and MAPK signaling pathways in LPS-activated mouse microglia BV2 cells. Our results demonstrated that 5Z-7-oxozeaenol suppresses the activation of the MAPK pathways, but has no effect on NF-κB pathway. NF-κB can be activated through a canonical or non-canonical pathway. The canonical (or classical) NF-κB signaling pathway is induced through tumor necrosis factor receptor 1, Toll-like receptors, interleukin-1 receptor, or T and B cell receptors. Subsequently, TAK1 is phosphorylated, leading to downstream IκBα phosphorylation, which mediates NF-κB activation (Guire et al., 2013). Furthermore, analogous to the phenomenon observed with p38MAPK inhibitors (Cohen, 2009). Inhibition of NF-κB by 5Z-7-oxozeaenol could lead to hyperactivation of the NF-κB pathway by a feedback mechanism that occurs in the mouse model and *in vitro*.

## Conclusion

The present study demonstrates that 5Z-7-oxozeaenol potently protects mice from EAE progression, decreases inflammatory responses and demyelination of the CNS, deactivates microglia and inhibits the secretion of pro-inflammatory cytokines by blocking the p38MAPK, JNK, and ERK signaling cascades. Our findings confirm that 5Z-7-oxozeaenol represents a potential novel drug that can be applied clinically to CNS autoimmune disorders. Other drugs targeting TAK1 might also provide a promising approach for modulating the course of the immune reaction in autoimmune demyelinating diseases and potentially other chronic inflammatory diseases.

## Author Contributions

SQ and PX conceived and designed all experiments in this study. LL established EAE mice model, performed H&E staining, LFB staining, immunohistochemistry, ELISA, and Western blots assays. XZ and WZ performed the behavioral experiment. HT carried out ELISA. SQ, LL, XZ, and PX analyzed the data. LL and SQ wrote the manuscript.

## Conflict of Interest Statement

The authors declare that the research was conducted in the absence of any commercial or financial relationships that could be construed as a potential conflict of interest.
